# Poly(I:C) induces intense expression of c-IAP2 and cooperates with an IAP inhibitor in induction of apoptosis in cancer cells

**DOI:** 10.1186/1471-2407-10-327

**Published:** 2010-06-24

**Authors:** Luc Friboulet, Claire Gourzones, Sai Wah Tsao, Yannis Morel, Carine Paturel, Stéphane Témam, Catherine Uzan, Pierre Busson

**Affiliations:** 1 Univ Paris-Sud, CNRS-UMR 8126 and Institut de Cancérologie Gustave Roussy, 39 rue Camille Desmoulins, F-94805 Villejuif, France; 2 Department of Anatomy, the University of Hong-Kong, Hong-Kong; 3 Innate Pharma, 117 Avenue de Luminy, F-13276 Marseille, France; 4 Univ Paris-sud and Institut de Cancérologie Gustave Roussy, cervico-facial surgery unit, 39 rue Camille Desmoulins, F-94805 Villejuif, France; 5 Univ Paris-sud and Institut de Cancérologie Gustave Roussy, gynecology unit, 39 rue Camille Desmoulins, F-94805 Villejuif, France

## Abstract

**Background:**

There is increasing evidence that the toll-like receptor 3 (TLR3) is an interesting target for anti-cancer therapy. Unfortunately, most laboratory investigations about the impact of TLR3 stimulation on human malignant cells have been performed with very high concentrations - 5 to 100 μg/ml - of the prototype TLR3 ligand, poly(I:C). In a previous study focused on a specific type of human carcinoma - nasopharyngeal carcinoma - we have shown that concentrations of poly(I:C) as low as 100 ng/ml are sufficient to induce apoptosis of malignant cells when combined to a pharmacological antagonist of the IAP family based on Smac mimicry.

**Methods:**

This observation prompted us to investigate the contribution of the IAP family in cell response to poly(I:C) in a variety of human malignant cell types.

**Results:**

We report a rapid, intense and selective increase in c-IAP2 protein expression observed under stimulation by poly(I:C)(500 ng/ml) in all types of human malignant cells. In most cell types, this change in protein expression is underlain by an increase in c-IAP2 transcripts and dependent on the TLR3/TRIF pathway. When poly(I:C) is combined to the IAP inhibitor RMT 5265, a cooperative effect in apoptosis induction and/or inhibition of clonogenic growth is obtained in a large fraction of carcinoma and melanoma cell lines.

**Conclusions:**

Currently, IAP inhibitors like RMT 5265 and poly(I:C) are the subject of separate therapeutic trials. In light of our observations, combined use of both types of compounds should be considered for treatment of human malignancies including carcinomas and melanomas.

## Background

Toll-like receptor 3, a membrane receptor of double strand RNAs, is a major effector of the immune response against viral pathogens at the cellular and systemic level. It is involved in early activation of NK and dendritic cells. It is also expressed in a wide range of non-immune cells where it plays a key role in the induction of interferon response [1]. TLR3 is frequently expressed by malignant cells of various types and there are several observations suggesting that it can be targeted for therapeutic purpose [2,3]. At least one clinical trial has shown a therapeutic benefit for breast carcinoma patients treated with the synthetic TLR3 agonist poly(A/U) [4]. On the other hand, several *in vitro *studies have reported apoptosis induction in malignant cells treated with the synthetic TLR3-agonist, poly(I:C). However, these results were obtained using very high concentrations of this agent in the range of 10 to 100 μg/ml [5-9]. Such concentrations are probably incompatible with doses of synthetic ligands acceptable for patient treatment. One of our previous study focused on nasopharyngeal carcinoma has opened news perspectives in this field [10]. Nasopharyngeal carcinoma or NPC is a human epithelial tumor whose malignant cells are latently infected by the Epstein-Barr virus (EBV). Using our experimental model of NPC, we could demonstrate that massive caspase-dependent apoptosis was induced in NPC cells by poly(I:C) at a low concentration (500 ng/ml) when it was combined to RMT 5265 (100 nM), a synthetic inhibitor of the IAP family of proteins [10].

Inhibitor of apoptosis proteins (IAP) are a class of regulatory proteins, with mainly anti-apoptotic properties, characterized by the presence of one to three domains known as baculoviral IAP repeat (BIR) domains [11]. Among these IAP proteins, X-linked IAP (XIAP) is a direct inhibitor of caspase activity. It is produced in large amounts in all cell types and is often regarded as a housekeeping protein [11]. Cellular IAP-1 (cIAP-1) and cIAP-2 have more complex regulatory functions, many of these functions involving their E3 ubiquitin-ligase activity [12-14]. Recent studies have emphasized their connection with TNF-receptor signaling and NF-kB activation [14-16]. They are expressed at various levels in cancer cells depending on the tumor type [10]. Second mitochondria-derived activator of caspase (Smac) is an endogenous antagonist of IAP protein [17]. In its dimeric form, Smac, via its AVPI tetrapeptide binding motif, binds the BIR domains of XIAP, c-IAP1 and 2. It causes proteasome-dependent degradation of c-IAP1 and c-IAP2 [17]. RMT 5265 is the prototype of a new class of anticancer drugs called Smac mimetics [18]. This polycyclic compound was designed for spatial mimicry of the AVPI motif of the Smac protein. It is cell permeable and specifically binds c-IAP1, c-IAP2 and XIAP, triggering rapid proteasome-dependent degradation of c-IAP1 and c-IAP2 [10,18]. It is also suspected to antagonize the functions of XIAP [18].

Our previous study on NPC cells provided the proof of principle 1) that synthetic TLR3 ligands could be active on malignant cells at much lower concentrations than previously reported (below 1 μg/ml); 2) that the IAP family of proteins was very important to modulate cell response to TLR3 stimulation and 3) that combinations of TLR3 ligands with IAP inhibitors were susceptible to provide a therapeutic benefit [10]. However NPC cells have unique biological features, for example a low frequency of p53 mutations, in addition to a latent EBV-infection in virtually 100% of the cases [19]. Therefore, it was important to investigate the role of IAPs in other types of malignant cells subjected to TLR3 stimulation. Using a panel of various human carcinoma and melanoma cell lines, we decided to address the following questions: 1) What is the influence of a TLR3 synthetic ligand on the status of the IAP proteins? 2) Can we enhance the pro-apoptotic effect of a TLR3 ligand by combination with an IAP inhibitor?

We report that the basal concentration of c-IAP2 is at a low level in a majority of malignant cell types, in constrast with our previous observations in NPC cells. However, stimulation of TLR3 by the synthetic ligand poly(I:C) induces a rapid, substantial and specific increase in c-IAP2 protein content, in all malignant cells tested. This increase is, at least to a large extent, explained by a transcriptional effect. When poly(I:C) was combined with the IAP inhibitor RMT 5265, we observed a cooperative effect in apoptosis induction and/or inhibition of clonogenic growth in a wide range of cancer cells.

## Methods

### Cell lines

The following malignant cell lines were used: HeLa (epithelial, cervix carcinoma); T1, SK29 and Rosi (melanomas); SKOV3 and IGR-OV1 (ovarian carcinomas); LNCaP (prostatic carcinoma) [16,20]. MRC5 cells are non-malignant human fibroblasts purchased from Biomerieux (Marcy l'Etoile, France). The NP69 cell line was obtained by SV40 infection of epithelial cells derived from a piece of non-malignant human nasopharyngeal mucosa [21]. NP69 cells were grown in KGF keratinocyte medium supplemented with 10% fetal calf serum (FCS). LNCaP, SK29, Rosi, IGR-OV1 and MRC5 cells were continuously grown in RPMI 1640 medium (Gibco-Invitrogen, Cergy Pontoise, France) supplemented with 10% FCS, SKOV3 cells were grown in Mc Coy's 5A medium (Gibco-Invitrogen) supplemented with 10% FCS and 2% sodium bicarbonate, T1 cells in RPMI 1640 medium supplemented with 10% FCS and 1% sodium pyruvate and HeLa cells in Dulbecco's modified Eagle medium (DMEM) (Gibco-Invitrogen) supplemented with 5% FCS.

### Preparation of cell suspensions from tumor biopsies

Fragments of tumor biopsies were obtained after written informed consent from two patients treated at the Institut de Cancérologie Gustave Roussy (Villejuif, France). Patient A, male, aged 42, had a well differentiated squamous cell carcinoma of the larynx, staged T2N0M0. Patient B, male, aged 52 had a differentiated squamous cell carcinoma of the oral cavity staged T4N2bM0. A few minutes after collection, these tumor fragments were minced in small pieces (1 to 2 mm diameter) and incubated sequentially 1) in a solution of trypsin (1.25 mg/ml)(Sigma, St Quentin Fallavier, France) in DPBS (Dulbecco Phosphate Buffered Saline - Invitrogen) at 4°C for 90 min with agitation; 2) then in a solution of collagenase (4 mg/ml)(type 2, Worthington) and DNAse (10 μg/ml)(Sigma) in RPMI culture medium with 20% fetal calf serum at 37°C for 3 hours. Tumor cells were then dispersed by energetic pipet aspirations and releases, washed, filtered through a 70 μm cell strainer, counted and seeded in 24-well culture plates at a density of 0.5 million/well.

### Treatments of cells with pharmacological reagents

The polycyclic C2-symmetric (40 carbon atoms) compound RMT 5265, which mimics the three-dimensionnal structure of the Smac/Diablo N-terminal tetrapeptide, has been previously described [18]. HS4044 or control agent, has a similar structure but is acetylated at a critical alanine group; it was used as a negative control [18]. Both reagents were dissolved in DMSO. Agonists of TLR3 (poly(I:C)) and TLR9 (type C CpG) were obtained from InvivoGen (Toulouse, France). Bafilomycin A1 (endosomal acidification inhibitor) and 2-Aminopurine (PKR inhibitor) were purchased from Sigma.

### RNA interference

Expression of proteins was knocked down using the following siRNA: TRIF (TICAM1) (1: HSS152364; 2: HSS152365; 3: HSS175528), TLR3 (1: HSS110815; 2: HSS110817), c-IAP2 (HSS100561), XIAP (HSS100565). These siRNA and a negative control (NS; ref. 1390109) were purchased from Invitrogen. Transfections were carried our using Oligofectamine (Invitrogen). The final concentration of siRNAs in the culture medium was 100 nM for 4 h and 50 nM for the next 44 h. The impact on protein expression was assessed by western blotting, 48 h or 72 h after the initiation of transfection.

### RNA extraction, reverse transcription and polymerase reaction

Total RNA was extracted from cultured cells with the RNeasy extraction kit (Qiagen, Valencia, CA). Total RNA (1 μg) was reverse-transcribed using the Protoscript First Strand cDNA Synthesis Kit (New England BioLabs, Ipswich, MA). Real-time PCR was performed in a 25 μl reaction volume, containing 25 ng of cDNA template, 10 pmol of each primer and 12.5 μl of TaqMan Universal PCR Master Mix (Applied Biosystems, Foster City, CA). The following sets of primers and probes were purchased from Applied Biosystems (TaqMan Gene Expression system): BIRC3 (HS00154109_m1), TLR3 (HS01551078_m1), TLR4 (HS00152939_m1), TLR9 (HS00370913_s1), and GAPDH (HS99999905_m1). Amplification reactions were performed in an Applied Biosystems Abi Prism 7000 Sequence Detection System. Data from RQ-PCR were analysed using the comparative CT method with GADPH as an endogenous reference.

### Cell Growth Assays

Clonogenic growth of various cell types was assessed by plating cells at low density in six well plates. Initial cell densities were established for each cell type on the basis of pilot experiments varying from 2000 (NP69) to 5000 (others cell types) per well in six well plates. After 2 to 4 weeks of culture, cell colonies were stained with a solution of crystal violet. Dried plates were then scanned and digitized to allow optical magnification and precise counting of cell colonies.

### Assessment of Apoptosis and Caspase Activation

Apoptosis was assessed quantitatively by determining the sub-G1 DNA content in ethanol-fixed cells, stained with propidium iodide and analyzed using a Becton Dickinson FACScalibur flow cytometer and the CellQuest Pro software. Alternatively, apoptosis was evaluated by the detection of the cleavage of poly (ADP-ribose) polymerase (PARP) by western blot analysis performed on total cell protein extracts. The activities of caspases-3/7 and caspase-8 were measured with the Caspases-Glo 3/7 and Caspases-Glo 8 Assay kits, respectively (Promega, Lyon, France). These assays are based on the cleavage of luminogenic substrates containing the amino-acid sequences Z-DEVD and Z-LETD, respectively.

### Cell protein extraction and western blot analysis

Proteins from cultured cells were extracted by lysis in RIPA buffer (50 mM Tris, 150 mM NaCl, 5 mM EDTA, 0.5% sodium DOC, 0.5% NP40, 0.1% SDS) supplemented with a protease inhibitor cocktail (Complete, Roche, Meylan, France). They were separated by SDS-PAGE and transferred to PVDF membranes (Immobilon, Millipore, Billerica, CA) by electroblotting at 4°C for 90 minutes at 90 V or overnight at 45 V. The antibodies used for western blotting were mouse monoclonal antibodies directed against human c-IAP2, XIAP and c-IAP1, obtained from BD Biosciences (reference 552782, 610763 and 556533, respectively; Le Pont de Claix, France). The other mouse monoclonal antibodies used were specific for FLICE-like inhibitory protein (FLIP) (Alexis Biochemical, ref. 804-428; San Diego, CA), PARP (Santa Cruz Biotechnology; ref. 53643; Heidelberg, Germany), TRIF (Cell Signalling, ref. 4596; Danvers, MA), caspase-8 (Cell Signaling, ref. 9746), TLR3 (R&D systems ref. MAB1487; Lille, France), β-actin (Millipore, Billerica, CA, ref. MAB1501) and tubulin-α (Sigma, ref. T5168). Blots were incubated with a secondary peroxidase-conjugated antibody and chemiluminescent detection was done using the Immobilon Western Chemiluminescent HRP Substrate (Millipore, Billerica, CA).

### Statistics

Results of real-time PCR, colony assays and caspase assays were given as the means +/- standard deviation (SD). Statistical significance was assessed using a two-tailed Student's t test for comparison of 2 experimental conditions. When making comparisons involving multiple experimental conditions we used the ANOVA test completed by the Tukey test (bilateral) in the XLSTAT software (confidence interval 95%).

## Results

### TLR3 transcripts are constitutively expressed in various types of human malignant cells

**Figure 1:**
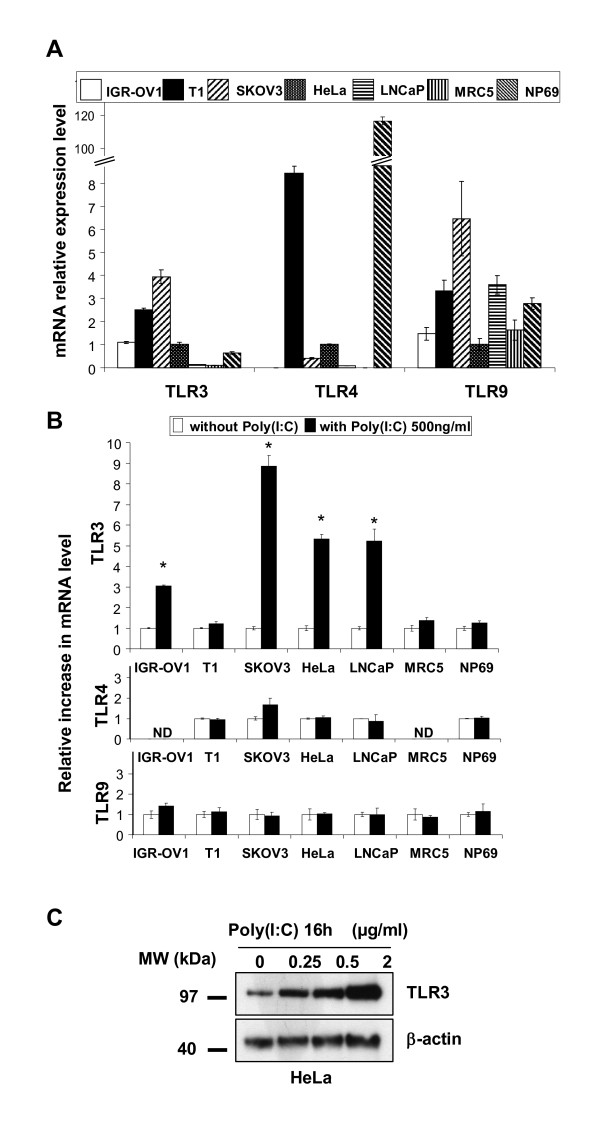
**Assessment of TLR 3, 4 and 9 messenger RNAs and TLR3 protein in a panel of human cell lines in basal conditions and under stimulation by poly(I:C)**. A) Relative expression levels of TLR3, 4 and 9 transcripts in basal conditions. Levels of mRNAs in HeLa cells were chosen as a reference and arbitrarily set at 1. B) TLR mRNA relative increase in expression under stimulation by poly(I:C) (500 ng/ml) for 16 h. For each cell type, the basal level of expression was taken as a reference and set at 1. C) Western blot detection of the TLR3 protein in HeLa cells treated for 16 h with increasing concentrations of poly(I:C)(from 250 ng/ml to 2 μg/ml). β-actin staining was used to control protein loading. Data presented in A), B) and C) are representative of at least two similar experiments. The stars indicate a statistical difference from respective controls (p < 0.05).

TLR3 expression has been reported in a wide range of human malignant cells [5,6,8]. To confirm this observation, TLR3 messenger RNAs were analyzed by quantitative RT-PCR in a series of human malignant cell lines including one melanoma (T1), two ovarian carcinomas (IGR-OV1 and SKOV3), one endocervical carcinoma (HeLa), one prostate carcinoma (LNCaP). Two human control cell lines were analyzed in parallel: a human diploid fibroblast cell line (MRC5) and an SV40-immortalized non-tumorigenic cell line derived from human nasopharyngeal epithelial cells (NP69). As shown in Figure 1A, transcripts of TLR3 were readily detected in all 5 malignant cell lines at a higher level than in non-tumorigenic MRC5 and NP69 cells. The distribution of TLR4 and 9 transcripts was quite different. There were homogeneous high levels of TLR9 transcripts in tumorigenic and non-tumorigenic cells and a more restricted expression of TLR4 with high levels in T1 and NP69 cells. In some experimental systems, double strand RNA has been shown to induce a positive feed-back on TLR3 expression [22]. We investigated whether the same positive feed-back mechanism could happen in our cell lines under treatment by poly(I:C) at 500 ng/ml for 16 h. As shown in Figure 1B, we observed almost no changes in TLR4 and TLR9 transcript levels. In contrast, TLR3 expression was enhanced more than 2 fold in 4 of the 5 malignant cell lines and only slightly modified in MRC5 and NP69. The increase in TLR3 messenger RNAs resulted in an increase in protein expression as shown in Figure 1C. In summary, TLR3 transcripts are abundant and highly inducible in various types of human malignant cells whereas they are at a low level and not inducible in two different types of human non-malignant cells.

### Poly(I:C) treatment increases c-IAP2 protein concentration in various types of human malignant cells

**Figure 2:**
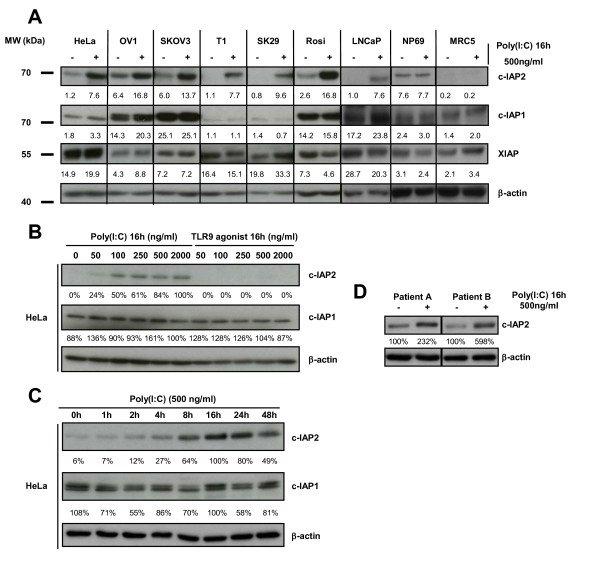
**Status of the IAP proteins in a panel of malignant and non-malignant cell types in basal conditions and under stimulation by the poly(I:C)**. A) Variations in IAP protein concentrations in basal conditions and after 16 h of stimulation by poly(I:C) 500 ng/ml. The c-IAP1, c-IAP2 and XIAP relative concentrations were assessed by iterative staining of blotted membranes. β-actin was used as a loading control for density measurements. B) Exploration of the dose-effects relationships in c-IAP2 induction in HeLa cells treated for 16 h by poly(I:C) (TLR3 agonist) or CpG DNA (TLR9 agonist) at concentrations increasing from 50 to 2000 ng/ml. The same membrane was stained successively with anti-c-IAP2, c-IAP1 and β-actin. C) Western blot analysis of HeLa cells treated with poly(I:C) 500 ng/ml for increasing time intervals from 1 h to 48 h. The same membrane was stained successively with anti-c-IAP2, c-IAP1 and β-actin. D) Increase in c-IAP2 protein expression in malignant cells directly explanted *in vitro *from fresh human tumors. Cells were prepared for short term primary cultures from tumor biopsy fragments obtained from 2 patients designated A and B. These patients were referred for Head and Neck squamous cell carcinomas (see Methods section). Cells were incubated with poly(I:C) immediately after tumor dispersion for 16 h and collected for protein extraction and western blot analysis of c-IAP2 and β-actin expression. Data presented in A), B) and C) are representative of at least two similar experiments.

In our previously published tumor model - nasopharyngeal carcinoma (NPC) - c-IAP2 cell concentrations were uniformly very high whereas c-IAP1 was consistently undetectable [10]. In contrast, we knew from preliminary experiments that the basal levels of c-IAP1 and c-IAP2 were highly variable in various types of non-NPC malignant cells. Therefore we decided to assess the status of several IAP proteins - c-IAP1, c-IAP2 and XIAP - in basal conditions and under treatment by poly(I:C). The panel of cell lines described in the previous experiment completed by two additional melanoma cell lines (SK29 and Rosi) was subjected to stimulation by poly(I:C), 500 ng/ml for 16 h. Then the cell concentrations of IAPs were compared by western blotting in the absence or in the presence of poly(I:C). As shown in Figure 2A, in basal conditions, c-IAP2 was at a low level of concentration in most malignant cell types. However, in the presence of poly(I:C), its concentration was substantially increased in all types of malignant cells with amplitudes varying from 2 (SKOV3) to 10 fold (SK29). In contrast c-IAP2 concentrations were not modified in NP69 and MRC5 (in the latter, c-IAP2 remained undetectable). Simultaneously, the levels of c-IAP1 and XIAP concentrations were not significantly modified except a 70% increase in XIAP concentration in the SK29 cell line. The dose-effect relationships and kinetics of the increase in c-IAP2 protein expression was investigated in HeLa cells. An increase in c-IAP2 protein concentration was detectable with concentrations of poly(I:C) as low 50 ng/ml; it was dose-dependent from 50 to 250 ng/ml and then reached a plateau (Figure 2B). Stimulation by a TLR9 agonist in the same range of concentrations had absolutely no effect on c-IAP2 concentration. c-IAP1 concentrations were not modified either by poly(I:C) or the TLR9 agonist. In terms of kinetics, the increase in c-IAP2 cell concentration was detectable between 4 and 8 hours with a peak between 16 h and 24 h (Figure 2C). Overall, these data demonstrate a specific increase in c-IAP2 cell concentrations induced by treatment with poly(I:C). This change is restricted to malignant cell types. In HeLa cells it is maximal between 16 h and 24 h and detectable with concentrations of poly(I:C) as low as 50 ng/ml. To provide evidence that the stimulatory effect of poly(I:C) on c-IAP2 expression was not restricted to malignant cell lines permanently propagated *in vitro*, we prepared tumor cells derived from Head and Neck squamous cell carcinomas and used them in short term primary cultures. These cells directly explanted from fresh tumors were treated with poly(I:C) (500 ng/ml) for 16 h. As shown in Figure 2D, we observed the same dramatic increase in c-IAP2 concentration.

### Poly(I:C) treatment specifically enhances c-IAP2 expression through TLR3 stimulation

**Figure 3:**
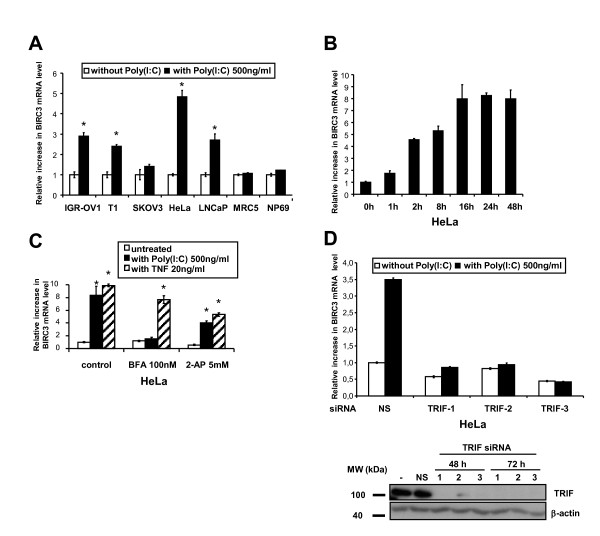
**Treatment by poly(I:C) increases c-IAP2 gene (*BIRC3*) transcription in human malignant cells. Inhibition by Bafilomycin A1 and knock-down of TRIF**. A) Cell concentrations of *BIRC3 *messenger RNAs are measured by quantitative RT-PCR in several types of malignant cells mock-treated or treated with poly(I:C) for 16 h at 500 ng/ml. Concentration of *BIRC3 *transcripts measured in basal conditions were chosen as a reference for each tested cell type and arbitrarily set at 1. B) Kinetics of the increase in the concentration of *BIRC3 *messenger RNAs in HeLa cells treated with poly(I:C) 500 ng/ml for increasing time intervals from 1 to 48 h. C) The influence of poly(I:C) on *BIRC3 *transcripts is neutralized by Bafilomycin A1, an inhibitor of endosome acidification and TLR3 signalling. Prior to RNA extraction, HeLa cells are incubated for two hours in the presence of poly(I:C)(500 ng/ml) or recombinant TNF α (20 ng/ml) in combination with Bafilomycin A1 (BFA) 100 nM or 2-aminopurin (2-AP) 5 mM or without additional compound (control condition). D) The induction of c-IAP2 by poly(I:C) in Hela cells is suppressed when the TRIF adaptor protein is knocked-down using 3 distinct specific siRNA whereas a non-specific (NS) RNA used as a negative control has no inhibitory effect. Upper panel: quantitative PCR assessment of the *BIRC3 *transcripts in the absence or in the presence of specific siRNAs. Lower panel: absence of TRIF protein expression detectable by western blot in Hela cells treated with specific siRNA (1, 2 and 3); in contrast TRIF expression is not altered by a negative control non-specific siRNA (NS). Data are representative of two similar experiments. The stars indicate a statistical difference from respective controls (p < 0.05).

**Figure 4:**
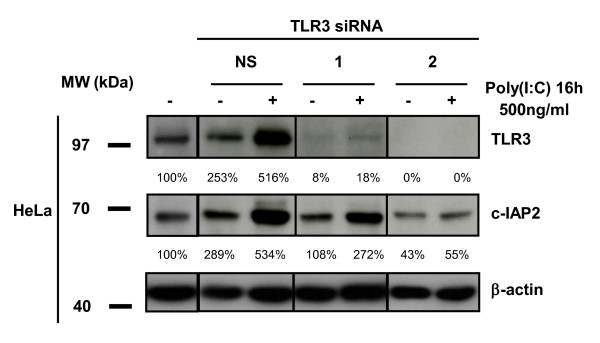
**Knocking-down TLR3 suppresses enhancement of c-IAP2 protein expression by poly(I:C)**. Western blot analysis of protein extracts from HeLa cells treated with poly(I:C) (500 ng/ml for 16 h) with or without pre-treatment with TLR3-specific and control (NS) siRNAs. The same membrane was stained successively with anti-c-IAP2, TLR3 and β-actin. Note that the control siRNA by itself enhanced TLR3 and c-IAP2 expression. This is consistent with previous observations and possibly related to the interaction of these small double-strand RNAs with the TLR3 itself regardless of their sequence [36]. In our transfection protocol siRNAs are at 100 nM for the first 4 h and 50 nM for the next 44 h. TLR3 inhibition was not complete with the siRNA n°1 which was apparently weaker than the siRNA n°2. However the siRNA n°1 was strong enough to prevent enhancement of TLR3 and c-IAP2 expression in the absence of poly(I:C). Data are representative of two similar experiments.

In order to understand how the cell concentration of c-IAP2 was increased by poly(I:C), we investigated its influence on the transcription of the c-IAP2 gene (*BIRC3*) in 5 malignant and 2 non-malignant cell types by quantitative reverse-PCR. Under stimulation by poly(I:C) 500 ng/ml, a statistically significant increase in cell concentrations of the *BIRC3 *transcripts was observed in 4 of 5 malignant cell types contrasting with virtually no increase in the non-malignant cell types (Figure 3A). In terms of kinetics, HeLa cells showed a dramatic increase in the amount of *BIRC3 *transcripts detected as early as 2 hours after the onset of the treatment by poly(I:C) with a plateau reached at 16 hours (Figure 3B). There are several known cellular receptors or sensors for double-strand RNA including TLR3 or PKR (protein kinase R) [23]. PKR is selectively inhibited by 2-aminopurine (2-AP) whereas TLR3 is inhibited by bafilomycine A1 (BFA). BFA is an inhibitor of the endosomal acidification pathway which prevents the adequate function of TLR3 within the endosomal compartment [23]. HeLa cells were stimulated by poly(I:C) (500 ng/ml) or TNF-α (20 ng/ml) which is a known inducer of c-IAP2 gene expression, in the absence or in the presence of inhibitors (BFA or 2-AP)(Figure 3C) [24]. BFA combined with poly(I:C) had a dramatic reducing effect on the amount of *BIRC3 *transcripts contrasting with almost no effects on *BIRC3 *transcription induced by TNF-α. The PKR inhibitor 2-AP had partial decreasing effects on the amount of *BIRC3 *transcripts under both poly(I:C) and TNF-α stimulation. The TRIF protein is a key adaptor molecule of TLR3 signal transduction whereas it is not required for signalling by other sensors of double strand RNAs like PKR [25] and RIG1 [26]. The TRIF protein was knocked out by RNA interference prior to the treatment of Hela cells with poly(I:C). As shown in Figure 3D, pre-treatment with TRIF-specific siRNAs resulted in a complete abrogation of the increase in *BIRC3 *transcription. Finally, to obtain a formal proof of TLR3 involvement, its expression was knocked-down in Hela cells, using 2 specific SiRNAs (Figure 4). One of them did not achieve a complete silencing of TLR3: in this context, an increase in c-IAP2 expression was still detected under treatment by poly(I:C). In contrast, when a complete extinction of TLR3 was obtained using a stronger siRNA, there was no detectable increase in c-IAP2 expression.

### An IAP inhibitor based on Smac mimicry (RMT 5265) cooperates with poly(I:C) in apoptosis induction

**Figure 5:**
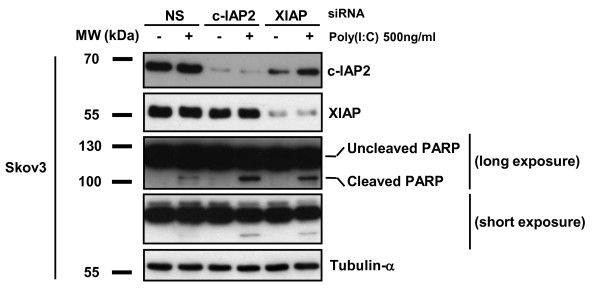
**Enhanced apoptosis in Skov3 ovarian carcinoma cells treated with poly(I:C) combined with knocking-down c-IAP2 or XIAP**. Skov3 cells were pre-treated with non-specific (NS), c-IAP2 or XIAP siRNAs with or without subsequent addition of poly(I:C). Cell protein extracts were subjected to western blot and stained successively for c-IAP2, XIAP, PARP and tubulin-α. PARP-cleavage was substantially enhanced by combination of poly (I:C) with c-IAP2 and XIAP siRNAs.

**Figure 6:**
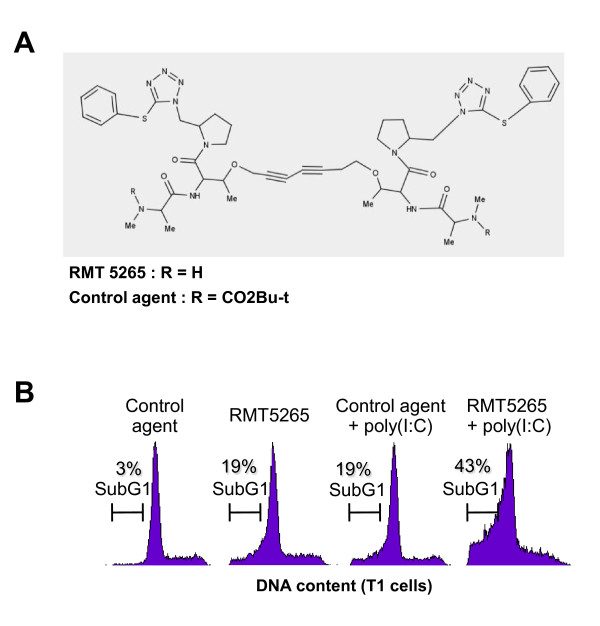
**Induction of apoptosis in T1 cells treated with poly(I:C) combined with a smac-mimetic (RMT 5265)**. A) Representation of the RMT 5265 molecule backbone drawn with MARVIN online software: http://atchimiebiologie.free.fr/marvin/doc/dev/oli.html. Substitution of one H on both alanines (R = CO_2_Bu-t) results in an inactive compound used as a negative control (HS4044). B) Flow cytometry assessment of the sub-G1 cell fraction in the T1 cell line treated with control compound or RMT 5265 (100 nM; 48 h) with or without poly(I:C)(500 ng/ml; last 16 h).

**Figure 7:**
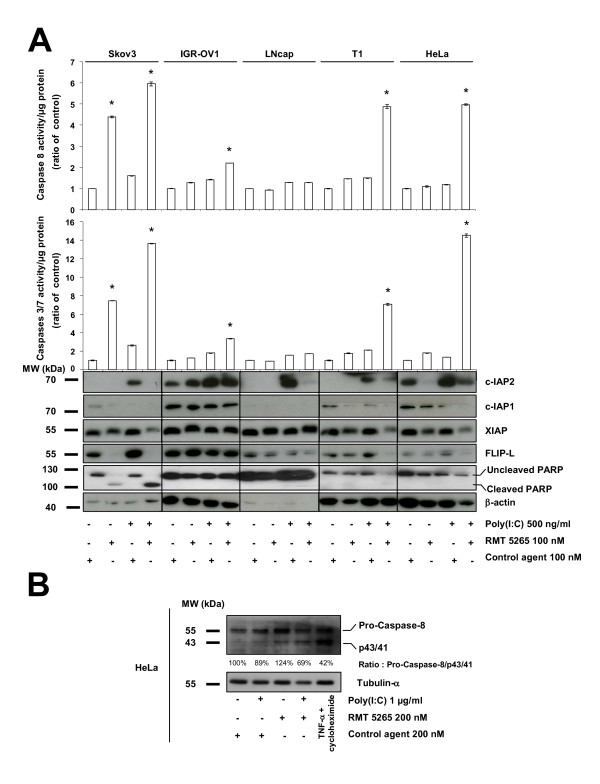
**Analysis of biochemical changes in human malignant cell lines treated with poly(I:C) combined to RMT 5265**. A) Cell samples representative of five different cell lines were subjected to the following treatments: control compound or RMT 5265 (100 nM for 48 h) with or without poly(I:C) (500 ng/ml; last 16 h). Caspase 3/7 (middle panel) and caspase 8 (upper panel) activities were measured in cell protein extracts using a chemiluminescent assay. c-IAP2, c-IAP1, FLIP-L and β-actin were detected in additional cellular extracts from the same experimental samples by western blotting using specific antibodies applied successively to the same blotted membrane (lower panel). The stars indicate a statistical difference from respective controls (p < 0.05). B) Western blot analysis of caspase-8 cleavage in HeLa cells treated with control compound or RMT 5265 (200 nM for 48 h) with or without poly(I:C) (1 μg/ml; last 16 h). In order to provide a positive control for caspase 8 cleavage, HeLa cells were treated with TNF-α (50 ng/ml) plus cycloheximide (2.5 μg/ml) for 6 h. The same blotted membrane was stained with anti-tubulin-α for protein loading control.

Poly(I:C) has been reported to induce apoptosis in several cell types at high concentrations [5-7]. On the other hand, the rise in c-IAP2 proteins and transcripts observed in our experiments suggested that poly(I:C) was also activating anti-apoptotic pathways, especially pathways involving the IAP family of proteins. We hypothesize that inhibition of c-IAP2 expression could unmask the apoptotic potential of TLR3 stimulation. To address this point Skov3 cells (derived from an ovarian carcinoma) were treated with siRNAs targeting either c-IAP2 or XIAP (Figure 5). An increase in PARP cleavage was observed when cells were treated with poly(I:C) following depletion of either c-IAP2 or XIAP, providing evidence that c-IAP2 as well as XIAP contribute to prevent TLR3-dependent apoptosis. To investigate whether these results could translate in a potential therapeutic strategy, we combined poly(I:C) with a pharmacological inhibitor of the IAP proteins, the Smac mimetic RMT 5265 (Figure 6A). Using the T1 melanoma cell line, we found additive effects of the combined treatment with a large increase in the sub-G1 fraction as shown by flow cytometry (Figure 6B). In the next experiment, cells from 5 tumor lines were treated with a single agent - either poly(I:C) or RMT 5265 - or by a combination of both agents. Total activities of caspases 3 and 7 were measured by a chemiluminescent assay providing an index of apoptosis. Massive activation of caspase 3/7 was obtained under the combined treatment in 3 out of 5 malignant cell lines (SKOV3, ovarian carcinoma, T1, melanoma and HeLa, Cervix carcinoma)(Figure 7A, middle panel). Caspase 3/7 activation was associated with degradation of the large PARP isoform (Figure 7A, lower panel). The effect of the combined treatment was obviously more than additive for caspase 3/7 activation in Hela cells. In SKOV3 cells, RMT 5265 by itself was sufficient for caspase 3/7 activation; however, its activity was increased two-fold when the cells were subjected to the combined treatment. A parallel increase in caspase 8 activation was noticed in all three cell types undergoing massive caspase 3/7 activation (Figure 7A, upper panel). The cleavage of caspase 8 was formally demonstrated by western blot analysis in Hela cells subjected to the combined treatment (Figure 7B). There was also mild apoptosis induction for IGR-OV1 with slight enhancement of caspase 3/7 activity (Figure 7A). LNCaP was the only cell line without any evidence of apoptosis induction under the combined treatment. Simultaneously c-IAP2, c-IAP1, XIAP and FLIP-L cell concentrations were assessed in these various experimental conditions by western blotting (Figure 7A, lower panel). Poly(I:C) stimulation induced a strong increase of c-IAP2 concentrations in all cell lines, as shown in previous figures. Treatment with RMT 5265 resulted in a dramatic reduction of c-IAP2 and to a lesser extent c-IAP1 when used alone as well as in combination with poly(I:C). This was observed in all cell lines except IGR-OV1. A reduction of FLIP-L was recorded in several experimental conditions, especially under the combined treatment. XIAP concentration was reduced in cells undergoing massive apoptosis under the combined treatment.

### Cooperation of poly(I:C) and RMT 5265 in the inhibition of clonogenic growth for various types of human malignant cells

**Figure 8:**
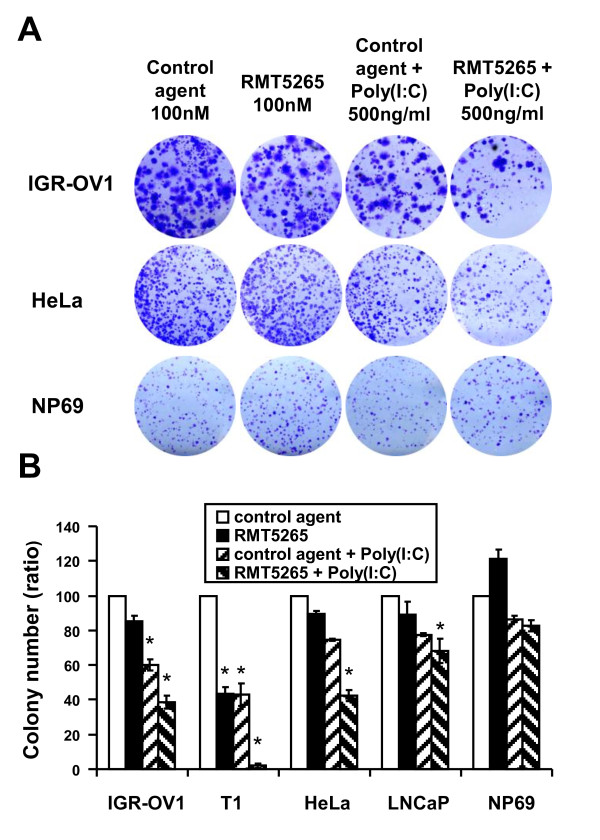
**Impact of poly (I:C) and RMT 5265 on the clonogenic growth of human cell lines**. Cells from IGR-OV1, T1, HeLa, LNCap and NP69 cell lines were seeded at low densities in 6 well-plates as reported in the Methods section. A) Plates were stained with crystal violet after 2 to 3 weeks of growth. B) Colonies were counted after scanning and digitalization of dried plates (Epson perfection 4990 scanner). For star-marked conditions, the differences in colony numbers with the control condition were statistically significant (p < 0.05).

To further explore the anti-tumor potential of the combination of poly(I:C) with RMT 5265, several cell types - IGR-OV1, T1, Hela, LNCaP and NP69 - were seeded at low density and grown in the presence of single agents or the combination of both (Figure 8). Poly(I:C) and RMT 5265 had virtually no effects on the non-tumorigenic NP69 cells. In contrast a highly significant reduction of clone numbers were obtained for cells undergoing massive apoptosis under the combined treatment, HeLa and T1. A similar reduction of clonogenic growth was observed for IGR-OV1 cells. Although there was no evidence of apoptosis induction in LNCaP, there was a mild but statistically significant reduction of its clonogenic growth under the combined treatment.

## Discussion and Conclusions

The aim of this study was to explore the contribution of the IAP family of proteins to the response of cells challenged by the synthetic TLR3 agonist poly(I:C). Our two main findings are a rapid and often dramatic increase in c-IAP2 protein concentration following poly(I:C) stimulation and a cooperative effect of poly(I:C) with the IAP inhibitor RMT 5265 in the induction of apoptosis and/or inhibition of clonogenic growth

The increase in c-IAP2 protein concentration triggered by poly(I:C) has several interesting characteristics. It is constant through all types of human malignant cells tested, including cells directly explanted from fresh tumors. So far we have found no exception to this type of response. Even in NPC cells which have very high constitutive levels of c-IAP2, we found that stimulation by poly(I:C) further increases its expression (Friboulet et al., 2008 and data not shown). However poly(I:C) does not induce any increase in c-IAP2 concentration in two types of non-tumorigenic human cell lines - NP69 and MRC5 - suggesting that this modification is restricted to fully transformed cells. Finally, the increase in c-IAP2 expression has a double specificity. With regard to the TLR family of receptors, it is induced by the TLR3 ligand poly(I:C) but not by ligands of other TLRs for example TLR-9 ligands (Figure 2B). With regard to the IAP family of proteins, although c-IAP1 and c-IAP2 expression are often co-regulated, poly(I:C) has no effect on c-IAP1 expression [27].

In all tumor cell lines but one, the increase in the c-IAP2 protein expression which occurrs under treatment by poly(I:C) is, at least to a large extent, related to an increase in the c-IAP2 gene (*BIRC3*) transcription. In this regard, the ovarian carcinoma cell line Skov3 is an exception. It has an increase in the expression of the c-IAP2 protein without a significant increase in the amount of the corresponding messenger RNA. The change observed at the protein level might result from a mechanism improving translation efficiency - "ribosome shunt" - as previously reported, precisely in a model involving c-IAP2 messenger and protein [28]. This hypothesis would deserve specific future investigations. In any event, we obtained compelling evidence that the changes in c-IAP2 expression observed in Hela cells treated with poly(I:C) are dependent on the activation of the TLR3/TRIF pathway. Indeed, the increase in *BIRC3 *transcription is completely abrogated by Bafilomycin A1 - a chemical agent known to block TLR3 signalling at the endosome level - as well as by knocking-down expression of the TRIF adaptor. Moreover, knocking-down TLR3 itself abrogates the increase in c-IAP2 expression. Currently, we have no idea whether NF-KB or interferon-β contribute to enhanced expression of the c-IAP2 gene downstream of TRIF. Nevertheless, in future studies, the increase in c-IAP2 expression is likely to provide a novel and sensitive index of malignant cell response to TLR3 stimulation.

Cell treatment combining RMT 5265 with poly(I:C) induces massive apoptosis in 3 of 5 malignant cell lines, with obvious synergy of the 2 compounds in two cases. Apoptosis induction is associated with decreasing cell concentrations of c-IAP2, c-IAP1, XIAP and FLIP-L. RMT 5265 like other smac mimetics is known to induce degradation of c-IAP2 and to a lesser extent c-IAP1 through ubiquitinylation and proteasome degradation [14,15]. Therefore we suspect that the decrease in c-IAP1 and c-IAP2 is an early drug-induced event with a possible causative role in the induction of apoptosis in T1 and HeLa cells. However, despite a dramatic decrease in c-IAP2 concentration and the absence of c-IAP1 expression in LNCaP cells, massive apoptosis is not induced by the combined treatment suggesting that other signalling events are required. c-IAP2 and c-IAP1 are not degraded in IGR-OV1 cells under treatment by RMT 5265. So far, in our experience, there is no other example of a cell line where c-IAP2 and c-IAP1 are resistant to degradation induced by RMT 5265. Nevertheless, we cannot exclude that they are functionally inactivated by RMT 5265 in IGR-OV1 cells. XIAP is usually not degraded by RMT 5265, therefore its decrease in SKOV3, HeLa and T1 cells is probably a consequence of massive apoptosis resulting from its digestion by executionary caspases [29]. So far we have no explanations for the decrease of Flip-L observed in several cell types under the combined treatment. In each of the 3 cell lines undergoing massive apoptosis, caspase 8 activation is prominent suggesting its critical role in the initiation of the process. Consistently, while this manuscript was in preparation, a recent publication has reported caspase 8 activation in melanoma cells subjected to a combination of poly(I:C) with a smac mimetic [30]. Involvement of caspase 8 is not surprising since RIP1, a major regulator of caspase 8 is under indirect control of TLR3 and c-IAP2. TLR3 stimulation activates RIP1 in a TRIF-dependent manner [31]. In the presence of c-IAP2, RIP1 is expected to activate the canonical NF-kB pathway whereas in the absence of c-IAP2, it will switch to caspase 8 activation [13,32-34]. Verification of this hypothetical mechanism will require further investigation.

The combination of poly(I:C) and RMT 5265 inhibits clonogenic cell growth in all 3 cell lines for which it induces apoptosis. In addition, clonogenic growth was also inhibited in the ovarian carcinoma cell line IGR-OV1 suggesting that the combined treatment has cytotoxic effects even in the absence of massive apoptosis induction. In contrast, the combined treatment has almost no effects on the non-transformed epithelial cell line NP69. This observation raises hope that synthetic TLR3 ligands combined to IAP inhibitors might have selective effects on malignant cells. One basis for this selectivity might be related to the status of TLR3 in malignant cells. In this regard it is interesting to observe a much greater positive feed-back of poly(I:C) on TLR3 expression in most malignant cells compared to MRC5 and NP69 (Figure 1B). In other words, TLR3 seems to be either more abundantly expressed or more "responsive" in fully transformed cells than in non-tumorigenic cells. A point that will need to be addressed in both biological and clinical studies. Assessing in cancer patients the selectivity and efficiency of TLR3 ligands combined to IAP inhibitors is a reasonable aim since both types of compound are already under phase II or III clinical trials [4,35].

## Abbreviations

BIRC3: baculoviral IAP repeat-containing 3; c-IAP: cellular Inhibitor of apoptosis protein; NPC: Nasopharyngeal Carcinoma; TLR3: toll-like receptor 3; Smac: Second Mitochondria-derived Activator of Caspases; XIAP: X-linked Inhibitor of Apoptosis Protein; TRIF: Toll/IL-1 Receptor domain-containing Adaptater-Inducing IFN.

## Competing interests

YM and CP are employed by Innate Pharma a company developing a TLR3 agonist. All other authors certify that they have no conflict of interest.

## Authors' contributions

LF and CG have performed most of the experiments and prepared the Figures. SWT has provided the NP69 cell line. YM and CP have shared they expertise regarding detection and silencing of the TLR3 protein. ST and CU have provided clinical samples. PB has designed the study and written most parts of the manuscript. All authors read and approved the final manuscript.

## Pre-publication history

The pre-publication history for this paper can be accessed here:

http://www.biomedcentral.com/1471-2407/10/327/prepub
